# Lessons from an active surveillance pilot to assess the pneumonia of unknown etiology surveillance system in China, 2016: the need to increase clinician participation in the detection and reporting of emerging respiratory infectious diseases

**DOI:** 10.1186/s12879-019-4345-0

**Published:** 2019-09-03

**Authors:** Nijuan Xiang, Ying Song, Yu Wang, Jiabing Wu, Alexander J. Millman, Carolyn M. Greene, Zhentao Ding, Jie Sun, Wei Yang, Guoxia Guo, Ruirui Wang, Ping Guo, Zhixing Ren, Lei Gong, Pengpeng Xu, Suizan Zhou, Dan Lin, Daxin Ni, Zijian Feng, Qun Li

**Affiliations:** 1 Chinese Center for Disease Control and Prevention, No. 155 Changbai Road, Changping District, Beijing, 102206 China; 2 US Centers for Disease Control and Prevention, Atlanta, GA USA; 3 Beijing Municipal Center for Disease Prevention and Control, Beijing, China; 4 Beijing Research Center for Preventive Medicine, Beijing, China; 5 Anhui Provincial Center for Disease Control and Prevention, Hefei, Anhui China; 6 Fuyang Prefecture Center for Disease Control and Prevention, Fuyang, Anhui China; 7 Lu’an Prefecture Center for Disease Control and Prevention, Lu’an, Anhui China; 8 The Second People’s Hospital of Fuyang City, Fuyang, Anhui China; 9 Lu’an People’s Hospital, Lu’an, Anhui China; 10 Changchun Prefecture Center for Disease Control and Prevention, Changchun, Jilin China; 11 Fujian Provincial Center for Disease Control and Prevention, Fuzhou, Fujian China

**Keywords:** Pneumonia of unknown etiology, Emerging respiratory infectious disease, Surveillance system evaluation, Novel influenza viruses, China

## Abstract

**Background:**

We sought to assess reporting in China’s Pneumonia of Unknown Etiology (PUE) passive surveillance system for emerging respiratory infections and to identify ways to improve the PUE surveillance system’s detection of respiratory infections of public health significance.

**Methods:**

From February 29–May 29, 2016, we actively identified and enrolled patients in two hospitals with acute respiratory infections (ARI) that met all PUE case criteria. We reviewed medical records for documented exposure history associated with respiratory infectious diseases, collected throat samples that were tested for seasonal and avian influenza, and interviewed clinicians regarding reasons for reporting or not reporting PUE cases. We described and analyzed the proportion of PUE cases reported and clinician awareness of and practices related to the PUE system.

**Results:**

Of 2619 ARI admissions in two hospitals, 335(13%) met the PUE case definition; none were reported. Of 311 specimens tested, 18(6%) were seasonal influenza virus-positive; none were avian influenza-positive. < 10% PUE case medical records documented whether or not there were exposures to animals or others with respiratory illness. Most commonly cited reasons for not reporting cases were no awareness of the PUE system (76%) and not understanding the case definition (53%).

**Conclusions:**

Most clinicians have limited awareness of and are not reporting to the PUE system. Exposures related to respiratory infections are rarely documented in medical records. Increasing clinicians’ awareness of the PUE system and including relevant exposure items in standard medical records may increase reporting.

**Electronic supplementary material:**

The online version of this article (10.1186/s12879-019-4345-0) contains supplementary material, which is available to authorized users.

## Background

China established the Pneumonia of Unknown Etiology (PUE) Surveillance System in 2004 for timely detection of emerging respiratory infectious diseases [1], and the system has played an important role in detecting human infections with novel avian influenza viruses including A(H5N1), A(H10N8), A(H9N2), and A(H5N6) [2–4]. Nevertheless, a 2007 evaluation identified persistent underutilization of the PUE surveillance system [5]. More recently, inconsistent reporting occurred during the initial 2013 outbreak of low pathogenic avian influenza (LPAI) A(H7N9) [henceforth A(H7N9)], prompting public health authorities to allow clinicians to report cases directly without expert consultation committee approval. This change resulted in the reporting of 1118 cases in 5 weeks compared with 1016 cases in the previous 10 years [6]. Laboratory and case investigation resources were quickly strained and reporting procedures reverted to those used prior to the outbreak [6]. As a result, case reporting subsequently decreased. A 2015 assessment of clinician and health administrator knowledge, attitude and practices related to PUE surveillance conducted within 43 healthcare facilities revealed a willingness to report PUE cases, but identified limited awareness of the PUE system, lack of understanding of the reporting process, and failure to follow the case definition [7].

To evaluate these gaps, we piloted a 3-month active surveillance program in two hospitals to 1) quantify the number of cases meeting the PUE case definition and the number reported and 2) to identify ways to improve the PUE surveillance system’s detection of respiratory infections of public health significance.

## Methods

### PUE reporting description and case definition

National guidelines [1] require all inpatient and outpatient healthcare facilities to report cases meeting the PUE case definition. Clinicians should report cases to an expert consultation committee, which after review of clinical and laboratory data determines whether to report the case to the PUE surveillance system [1]. If a case is reported to the PUE system, the local center for disease control and prevention (CDC) will conduct a field investigation, collect respiratory specimens and send them to a national influenza surveillance network laboratory for testing of avian influenza viruses and, if associated with clusters of respiratory disease or relevant travel history, testing of Severe Acute Respiratory Syndrome Coronavirus (SARS-CoV) and Middle East Respiratory Syndrome Coronavirus (MERS-CoV).

A PUE case is defined as an illness of unknown etiology with 1) axillary temperature > 38 °C, 2) radiographic pneumonia, 3) low or normal leukocyte count or low lymphocyte count during the early stages of disease, and 4) no improvement or worsening symptoms after 3–5 days of antimicrobial treatment per clinical guidelines [1].

### Evaluation sites

Participating hospitals were selected based on four criteria: if the facility 1) admitted at least 200 patients per month with a discharge diagnosis of pneumonia during February through May 2013–2015; 2) used an electronic Hospital Information System; 3) demonstrated willingness and capacity to collaborate with both national and local CDCs and 4) was located within one of China’s 21 of 31 provinces with previously identified H5N1 and/or H7N9 human cases.

We selected two tertiary hospitals in Anhui Province: the Second People’s Hospital of Fuyang City, a 1400-bed facility, which from February through May 2013–2015, admitted an average of 231 pneumonia patients per month, and Lu’an City People’s Hospital, a 2300-bed facility, which over the same time period admitted an average of 252 pneumonia patients per month. Fuyang Hospital, an infectious disease hospital, had experience treating human infections with avian influenza, while Lu’an Hospital, a general hospital, did not.

### PUE case screening and enrollment

After reviewing the hospital information systems, the evaluation team developed a list of 56 admission diagnoses that captured the majority of acute respiratory infections (ARI) (Additional file 1).

**Fig. 1:**
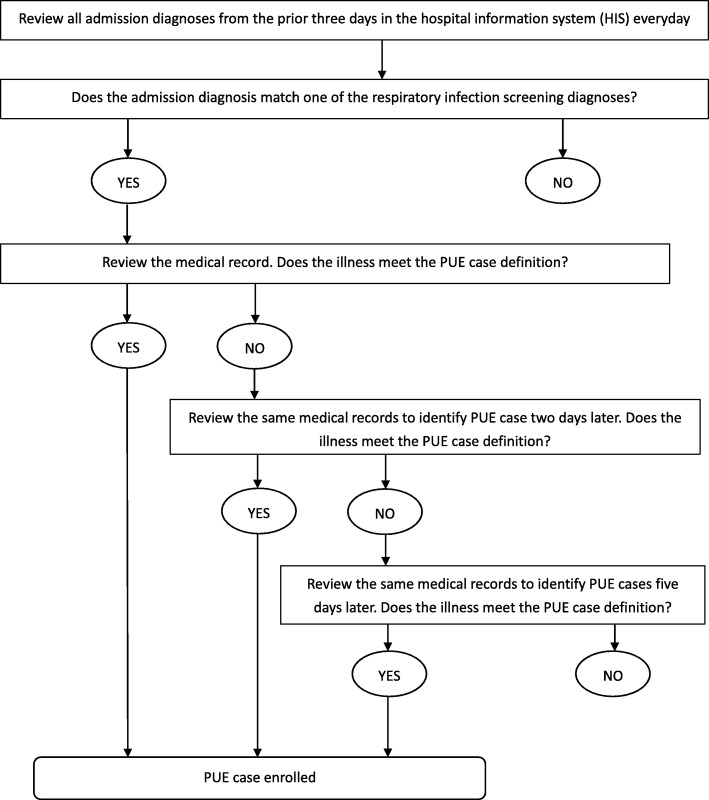
PUE case enrollment in this study

Every day (including weekends) from February 29 through May 29, 2016, a designated, trained surveillance officer in each hospital 1) reviewed the hospital admission registry database to screen all admission diagnoses from the prior 3 days for diagnoses from the screening list (Additional file 1); 2) reviewed admission medical records with a matching diagnosis to identify and enroll patients with illnesses meeting the PUE case definition; 3) 2 days later, conducted a second medical record review for patients not enrolled during the first review to identify and enroll patients with illnesses newly meeting the case definition (for example, patients with no improvement or worsening symptoms after 3–5 days of antimicrobial treatment per clinical guidelines); and 4) 5 days later, conducted a third review of records for patients not enrolled during the first two reviews to enroll any remaining patients meeting the case definition. [Fig. 1].

### Medical record review

For patients with illnesses meeting the PUE case definition, the surveillance officer used a standard questionnaire to collect information from the hospital information system related to demographics and, if available, epidemiological risk factors, including exposures to poultry, patients with similar symptoms, and travel history.

### Case investigation

Surveillance officers followed the PUE surveillance protocol [1] to investigate enrolled PUE case-patients. Surveillance officers conducted face-to-face patient interviews using a standard questionnaire to collect the same information described in the medical records review section above to determine both accuracy and completeness of medical records.

### Specimen collection and testing

Surveillance officers collected throat swabs from all identified PUE case-patients per the surveillance protocol [1]. Specimens were transported to the local CDC laboratory per standard procedures and tested for influenza viruses using real time reverse transcription polymerase chain reaction (rRT-PCR). If identified PUE case-patients were part of a cluster of epidemiologically-linked respiratory illnesses, specimens would also be tested for SARS-CoV and MERS-CoV. If a case-patient reported travel history to the Middle East, specimens would be tested for MERS-CoV.

### Assessing reporting procedures

**Fig. 2:**
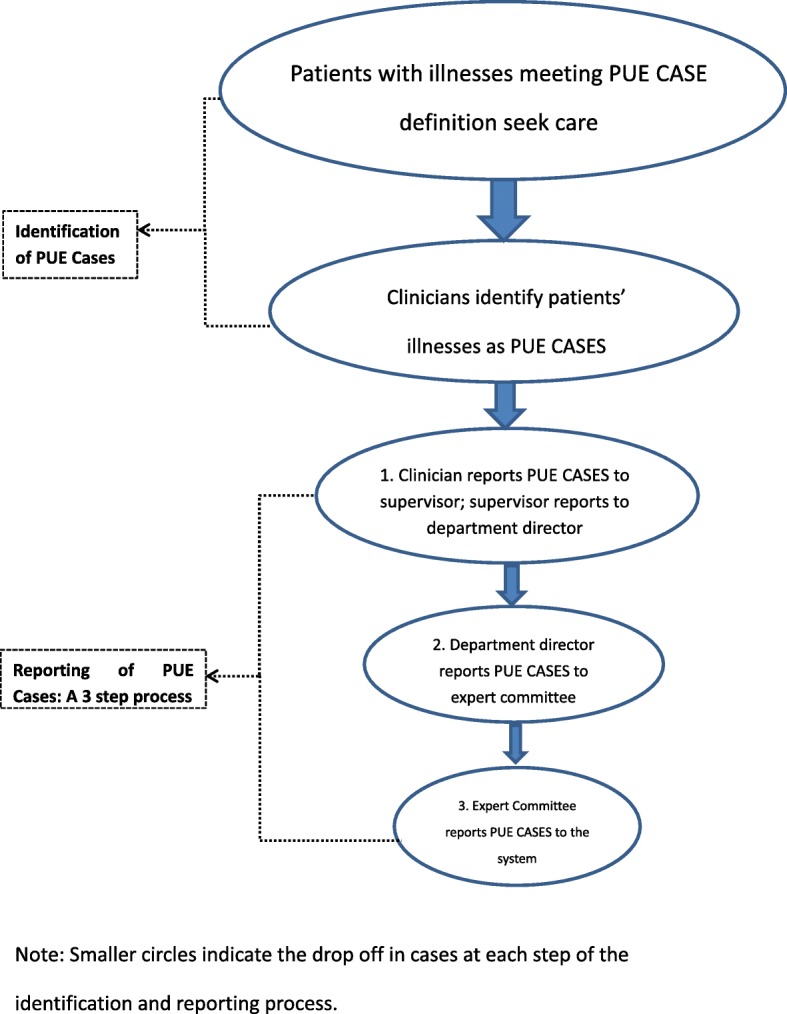
Schematic of the identification and reporting of PUE cases in China (with a 3-step reporting system)

The PUE surveillance protocol describes a three-step procedure for reporting cases to the PUE system: 1) clinicians report identified PUE cases to their supervisor; if the supervisor concurs, the case is reported to the director; 2) the director determines whether to report the case to an expert consultation committee which usually includes specialists from the respiratory medicine department, the radiology department, and infection control; and 3) the expert committee determines whether to report the case to the PUE system. [Fig. 2].

**Fig. 3:**
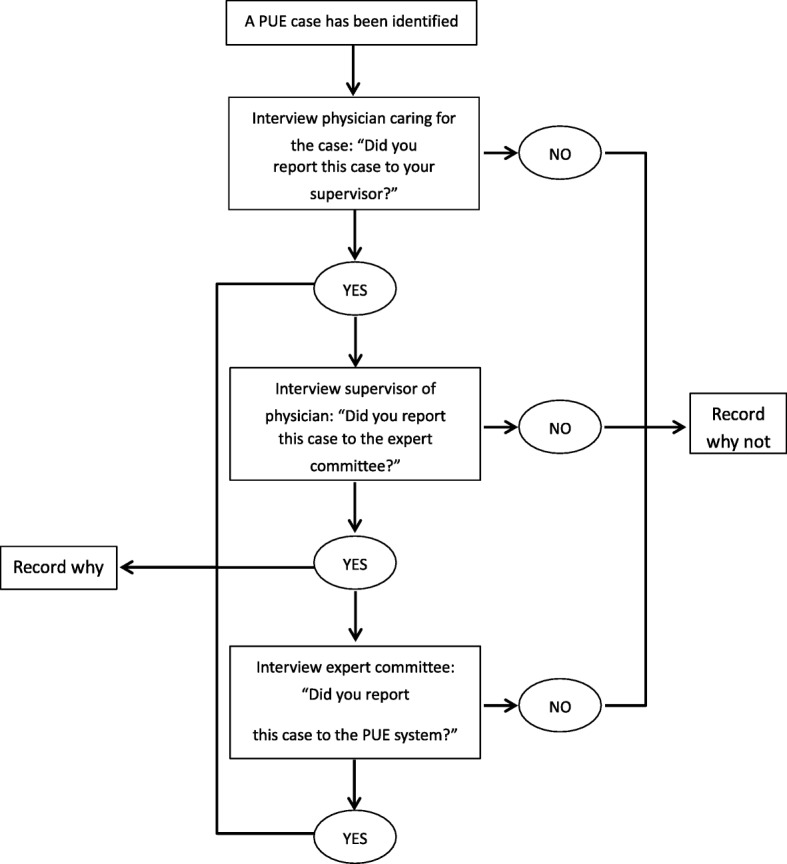
Assessment of PUE cases reporting process

In our evaluation, after patients with illnesses meeting the PUE case definition were enrolled, surveillance officers interviewed all clinicians who had primary medical responsibility for these patients. If the case were reported to the clinician’s supervisor, the surveillance officer also interviewed the supervisor and a representative member of the expert committee. [Fig. 3] Surveillance officers used a standard questionnaire to collect 1) demographic and occupational information about the clinician being interviewed and, when applicable, 2) demographic and occupational information about the senior clinicians who received the case report, and 3) reasons for reporting or not reporting PUE cases.

### Data analysis

First, we described the number, proportion, ward distribution, and testing results of PUE cases identified by surveillance officers. Wilson score was used to calculate 95% confidence intervals (CI) for proportions. Second, we described and compared epidemiologically-linked exposures documented by clinicians in the medical records and used chi-square tests to compare differences in the frequency of exposure histories collected by surveillance officer interviews. Third, we described the number of PUE cases reported at each of the tiers of the three-step reporting process. Finally, we described factors associated with clinicians’ reporting or not reporting PUE cases.

## Results

### Demographics and hospital wards of PUE cases identified

During the evaluation period, surveillance officers reviewed 11,203 hospitalization registrations, and screened 2619 patients with ARI admission diagnoses. Of those 1443 were from Lu’An Hospital and 1176 were from Fuyang Hospital. Among all ARI patients, 335 (13%) had illnesses that met the PUE case definition. The proportions of ARI cases meeting the PUE case definition in each of the two hospitals were similar [12% (180/1443) vs. 13% (155/1176), *P* = 0.59].

Among the 335 PUE case-patients identified, 60% were male, and 39% were aged 0–4 years; most were from the pediatrics (50%) and pulmonary (30%) departments. [Table 1]. The proportion of ARI admissions meeting the PUE case definition was highest in the ICU (48%), followed by the infectious disease (19%), tuberculosis (16%), pediatrics (13%) and pulmonary (10%) departments.

**Table 1:** Among all acute respiratory infection (ARI) admissions meeting screening criteria, distribution of pneumonia of unknown etiology (PUE) cases and non-PUE cases by ward, two hospitals, February – May 2016

Wards	ARI admissions in department (*N* = 2619) No. (%)	ARI admissions matching PUE case definition in Department (*N* = 335) No. (%)
Department of Pediatrics	1274 (49)	167 (50)
Respiratory Medicine	964 (37)	101 (30)
Tuberculosis Department	134 (5)	22 (6)
Infectious Diseases Department	89 (3)	17 (5)
ICU	29 (1)	14 (4)
Internal Medicine Emergency Room	58 (2)	7 (2)
Internal Medicine	48 (2)	5 (1)
Other Departments	23 (1)	2 (1)

### Laboratory results

Oral throat swabs, collected within 24 hrs of PUE case enrollment, were tested from 311 (93%) of the identified PUE case-patients. Of the 311 specimens, 18 (6%) were positive for seasonal influenza viruses and none were positive for avian influenza viruses. None met SARS-CoV or MERS-CoV testing criteria.

### Exposures among PUE case-patients

Of PUE case-patients with laboratory results, 56% (174/311) had documented exposures; 48% had documented histories related to contact with water potentially infected with parasites which is recorded as standard practice within the “personal history” section of the medical record in China. Other exposures which were intermittently documented in the 311 medical records included animal exposure (6%), contact with persons with respiratory illnesses (5%), exposure to healthcare facilities caring for patients with respiratory illnesses (10%), and any travel history (17%). In addition, 289 (93%) records documented occupation, a required item in the demographic section of the medical history, and identified two PUE case-patients as healthcare workers [Table 2].

**Table 2:** Comparison of epidemiological linked exposures of enrolled PUE cases reported to the surveillance officer versus documented in the medical record

Relevant Exposures	Surveillance Officer Interviewed Patients (*N* = 303)		Documented in Medical Record by Clinicians (*N* = 311)			p value
	Yes N (%, 95 CI*)	No N	Not Documented N(%, 95 CI)	Documented “Yes” N	Documented “No” N	
Had animal exposure** in 14 days before illness onset	62 (20, 16–25)	241	292 (94, 91–96)	0	19	0.03
Had close contact with similar cases of respiratory diseases	58 (19, 15–24)	245	296 (95, 92–97)	9	6	< 0.01
History of seeing a doctor in a medical institution with respiratory communicable diseases cases	58 (19, 15–24)	245	281 (90, 87–93)	13	17	< 0.01
History of traveling to or living in areas of novel respiratory epidemics	0	303	258 (83, 78–87)	0	53	–
Case works with poultry/livestock	3 (1, 0.3–2.9)	300	22 (7, 5–10)	2	287	1.00
Case is health care worker	3 (1, 0.3–2.9)	300	22 (7, 5–10)	0	289	0.25
Case is laboratory staff	0 (0, 0–1.3)	303	22 (7, 5–10)	2	287	0.24
Any contact with parasites-infected water	Not asked	Not asked	137 (44, 38–50)	0	174	–

*CI refers to Confidence Interval

**Exposure to poultry, pigs, et al.

Among the 303 enrolled PUE case-patients interviewed by surveillance officers, 131 (43%) had at least one exposure relevant to respiratory infections of public health significance, including animal exposure, contact with similar cases of respiratory disease, travel to/living in areas of novel respiratory epidemics, and occupational exposures. In the 14 days before illness onset, 3 (1%) had occupational exposure to poultry/livestock, 3 (1%) were medical staff, 62 (20%) had animal exposure (“exposure to poultry, pigs, etc.”), 58 (19%) had close contact with persons with similar respiratory disease symptoms, and 58(19%) had exposure to a healthcare facility caring for patients with respiratory illnesses [Table 2].

Although the relevant respiratory infectious disease exposures identified in the medical record and the surveillance officer interview were the same in > 85% for all exposures analyzed, there were discrepancies. Occupational exposures, exposures to persons with similar respiratory symptoms, and animal exposures were identified less frequently through medical records compared to surveillance officer interviews [Table 2].

### Clinician knowledge of PUE surveillance system

None of the 37 clinicians interviewed from Lu’an Hospital reported knowledge of the PUE surveillance system compared with 24 (55%) of the clinicians from Fuyang Hospital. At Fuyang Hospital, knowledge was highest among clinicians with > 10 years (100%) and 5–9 years of work experience (84%) and lowest among those with < 5 years of work experience (23%).

### PUE case reporting

None of the 335 patients meeting the PUE case definition were reported to the surveillance system. During the 307 interviews with 81 clinicians, the most common reasons clinicians cited for not reporting included: being unaware of the PUE surveillance system (76%), not understanding the PUE case definition (53%), and not accepting the PUE case definition (22%) [Table 3].

**Table 3:** Reasons listed for not reporting pneumonia of unknown etiology (PUE) cases, by hospital, 2016

Why the responsible clinician did not report the PUE case	The Second Hospital of Fuyang City (*N* = 151) n(%, 95 CI*)	The People’s Hospital of Luan City (*N* = 156) n(%, 95 CI)	Total (*N* = 307) n(%, 95 CI)
Was not aware of PUE surveillance system	76 (50, 42–58)	156 (100, 98–100)	232 (76, 70–80)
Did not understand the PUE case definition	6 (4, 2–8)	156 (100, 98–100)	162 (53, 47–58)
In their assessment, the patient’s illness did not meet the PUE case definition	68 (45, 37–53)	0 (0, 0–2.4)	68 (22, 18–27)
Wanted to treat the patient longer prior to reporting	16 (11, 7–17)	0 (0, 0–2.4)	16 (5, 3–8)
The patient was transferred to another hospital or ward	6 (4, 2–8)	0 (0, 0–2.4)	6 (2, 1–4)
When a case is reported, the laboratory results are returned too late such that the testing is not helpful for diagnosis and treatment	2 (1, 0.4–4.7)	0 (0, 0–2.4)	2 (1, 0.2–2.3)
Thought the reporting procedure was complex	1 (1, 0.1–3.7)	0 (0, 0–2.4)	1 (0.3, 0.06–1.83)
Wanted to report only after seeing a relevant lab result, such as H5N1 avian influenza	1 (1, 0.1–3.7)	0 (0, 0–2.4)	1 (0.3, 0.06–1.83)

*CI refers to Confidence Interval

Although none were reported to the national system, a clinician reported one PUE case with acute respiratory distress syndrome (ARDS) and possible viral pneumonia to his supervisor. Due to disease severity, the lack of a diagnosed pathogen, and no improvement on treatment, the supervisor reported the case to the director, and experts within the ward concluded that the illness met the PUE case definition. They reported the case to the hospital’s expert committee, which reported to the hospital’s department of disease control, which reported the case to the local CDC and sent specimens for laboratory testing. The test results were negative for influenza viruses (and the patient did not meet criteria for SARS or MERS testing); however, this case was not reported to the national PUE system despite meeting the case definition.

## Discussion

From February 29 through May 29, 2016, we conducted active surveillance in two hospitals and found that 13% of all patients admitted with ARI met the PUE case definition. None of the respiratory specimens tested were positive for avian influenza. Only one PUE case was reported to the local CDC; however, it was not reported to the national system because the specimen tested negative for avian influenza virus.

Our findings raise questions about the feasibility of using the existing PUE case definition to identify respiratory infections of public health significance. Extrapolating our results, if clinicians reported all illnesses meeting the PUE case definition from China’s more than 20,000 hospitals, the number of PUE cases identified would be in the hundreds of thousands per year. Such numbers would overwhelm the public health system’s capacity for laboratory testing and epidemiologic investigations. The impracticality of the existing PUE case definition is supported by both a prior study which found that 29% (442/1506) of community-acquired pneumonia diagnoses met the PUE case definition [8] and by the PUE surveillance experience in 2013 when streamlined reporting procedures led to a surge in cases that quickly strained response efforts [6]. Modifying the system to decrease the number of cases that meet the PUE case definition but are not emerging respiratory infections of public health significance would increase the system’s feasibility, acceptability, and usefulness.

The extent of under-reporting in this PUE assessment far exceeds the estimated 4–23% under-reporting of cases of notifiable diseases identified during 2005–2015 in evaluations of the China National Notifiable Disease Reporting System for 39 notifiable diseases, for which reporting is required by law [9–11]. During our evaluation, none of the 335 identified PUE cases was reported to the national system. One clinician reported one PUE case appropriately, but the expert committee did not appropriately follow final reporting procedures by reporting the case to the PUE system.

The two most common reasons clinicians cited for not reporting PUE cases to the system were not having knowledge of the PUE system and not understanding the PUE case definition. Knowledge varied by hospital, with > 50% of clinicians from Fuyang Hospital reporting knowledge of the PUE system compared with none from Lu’An Hospital. This difference may be explained by Fuyang Hospital’s specialization in infectious disease and its recent experience treating one avian influenza A(H5N1) virus infection and one avian influenza A(H7N9) virus infection in 2006 and 2014 respectively. Lu’An Hospital, a general hospital, had no recent experience treating infections with avian influenza virus. PUE surveillance system knowledge also varied significantly by clinicians’ years in practice. China CDC conducted intensive, national clinician training on the PUE system when the system was established and on-going trainings during the H5N1 outbreaks through 2008. In 2007, the Ministry of Health required training on the new protocol of Surveillance, Investigation and Management for PUE for clinicians at all medical institutions [12], however, national PUE-related training has not occurred since 2008. Therefore, clinicians with < 5 years work experience have not received systematic, in-depth training on PUE surveillance. Another recent study among hospital clinicians in Beijing found that new clinicians knew little about key public health concerns, such as infection control within healthcare facilities, without having received formal training [13]. These findings highlight the importance of training clinicians on public health surveillance systems, reporting requirements and other key public health topics both as they enter the workforce and as refresher courses to improve their capacity to identify and report emerging and re-emerging infectious diseases.

This evaluation found that clinicians did not document respiratory infection-related exposures. During the interviews, 20% identified PUE case-patients reported recent exposures to animals and 20% reported exposures to patients with similar illnesses, yet these exposures were rarely documented in the medical record. These findings suggest that clinicians may not routinely assess respiratory infection-related exposures. About half of the medical records from identified PUE cases documented any contact with parasites-infected water, because asking about “any potential exposure to parasite infected water” is a routine practice when completing the “personal history” section of the medical record [14]. This finding suggests that clinicians are more likely to ask about specific exposures when they are part of routine practice as opposed to exposures that may only be asked intermittently if not part of routine practice. The widespread use of electronic medical records in China provides an opportunity for prompting clinicians to ask about certain exposures relevant to infectious diseases that can be documented in a standardized way in patient’s medical record. By developing a checklist within the electronic medical record with questions related to exposures relevant to emerging respiratory diseases such as live poultry and swine for priority use in inpatient wards in China, clinicians would be prompted to routinely ask about these exposures. This may in turn improve detection and reporting of emerging respiratory infections. First, increasing documentation of relevant exposures may facilitate the addition of more specific epidemiologic criteria to the PUE case definition to reduce the number of cases meeting the case definition that are not infections of public health concern. Second, clinicians who identify concerning exposures in patients with respiratory diseases may be more likely to report these cases to the PUE system. Finally, since the majority of hospitals in China now use electronic medical records system, if exposure data were collected systematically as part of these systems, it is possible that cases with relevant epidemiological data could be automatically flagged for reporting. Further investigations would be needed to assess these modifications to see if they have a positive impact on case reporting without overwhelming the PUE system.

## Limitations

This evaluation is subject to several limitations. First, the assessment occurred when there were no local reports of human infection with avian influenza virus. PUE case identification and reporting may increase during outbreak periods. However, in 2017, during the 5th epidemic of influenza A(H7N9) which had the largest number of human infections to date, Fuyang Hospital did not report any cases to the national system despite sending 35 potential PUE case specimens to local CDC for testing, of which two were positive for influenza A(H7). During this same period, Lu’an Hospital did not send any potential PUE case specimens to local CDC for testing, nor did it report any cases to the national PUE system. Lu’an Hospital did, however, report one confirmed H7N9 case to China CDC directly. Second, the reporting practices within these two hospitals may not represent reporting practices throughout China. Finally, the screening admission diagnosis list may not have captured all PUE cases [Additional file 1].

## Conclusions

Our findings suggest that most clinicians are not reporting cases to the PUE surveillance system. If clinicians were to report all cases meeting the PUE case definition, the large number reported would likely overwhelm the public health system’s capacity for laboratory testing and case investigation. Of those reported, the vast majority of cases would not be emerging infections of public health concern. Our findings lead to several recommendations that may increase the specificity of the PUE case definition, increase clinician participation in the PUE system, and contribute to the early detection of emerging respiratory infections in China. 1) Modifying the existing PUE case definition by adding relevant exposure history may improve the specificity, feasibility and utility of case reporting. 2) Including exposure items related to emerging respiratory infectious diseases in the standard infectious disease history documented in medical records may increase the likelihood that clinicians will assess exposure histories relevant for emerging respiratory infections. Including these items in the respiratory diseases department, the pediatric department and the intensive care unit, where PUE cases are more common, may be most useful. 3) Providing clinicians with frequent public-health related training and communications will ensure that clinicians are aware of public health reporting requirements. A multi-pronged approach to incorporating public health practice into clinical settings may include: offering training to clinicians as they enter the workforce followed by annual refresher courses, posting public-health related updates and notices in clinical areas, incorporating public health guidance into hospital policies, engaging clinicians in the development of clinically-appropriate and easy-to-apply case definitions, and regularly sharing local and national public health data of interest with clinicians to highlight the public health importance of their work.

## Additional file

Additional file: 1: Screening admission diagnoses list. (DOCX 45 kb)

## Data Availability

All data generated or analysed during this study are included in this published article [and its supplementary information files]. The datasets generated and/or analysed during the current study are not publicly available due to the datasets containing personally identifiable information used for public health surveillance purposes. Requests for data can be directed to the corresponding author.

## Abbreviations

ARI: Acute Respiratory Infection
CDC: Center for Disease Control and Prevention
CI: Confidence Intervals
LPAI: Low Pathogenic Avian Influenza
MERS-CoV: Middle East Respiratory Syndrome Coronavirus
PUE: Pneumonia of Unknown Etiology
rRT-PCR: real time Reverse Transcription Polymerase Chain Reaction
SARS-CoV: Severe Acute Respiratory Syndrome Coronavirus
