# Supplementary material for: Lessons from an active surveillance pilot to assess the pneumonia of unknown etiology surveillance system in China, 2016: the need to increase clinician participation in the detection and reporting of emerging respiratory infectious diseases
Source: BMC Infect Dis. 2019 Sep 3;19:770. doi: 10.1186/s12879-019-4345-0 (PMC6724368; doi:10.1186/s12879-019-4345-0)
Supplement: Supplementary file 1 — Screening admission diagnoses list. (DOCX 45 kb) [file 12879_2019_4345_MOESM1_ESM.docx]

**Additional file 1**

Screening admission diagnoses list

1. Asthmatic suffocating pneumonia
2. Acute Respiratory Distress Syndrome (ARDS)
3. Chronic obstructive pulmonary disease combined with infection
4. Viral Pneumonia
5. Asthematoid bronchopneumonia
6. lobar pneumonia
7. Neonatal infection
8. Fever of Unknown Origin
9. Febrile seizures
10. Lung cancer with infections
11. Pulmonary infection
12. Tuberculosis associated with infection
13. Pneumonia
14. Pneumonia accompanies pleural effusion
15. Infective fever
16. [Respiratory tract infection](javascript:;)
17. Respiratory failure
18. Acute asthmatic bronchitis
19. Acute laryngotracheitis
20. Acute capillary bronchitis
21. Acute upper respiratory infection
22. Acute bronchitis
23. Interstitial pneumonia
24. Pneumonia in both lungs
25. Infections in both lungs
26. Interstitial fibrosis with infection in both lungs
27. Chronic bronchitis
28. Acute exacerbation of chronic bronchitis
29. Chronic obstructive pulmonary disease with acute lower respiratory tract infection
30. Acute Exacerbation Chronic obstructive pulmonary disease(AECOPD)
31. Chronic bronchitis associated with infection
32. Chronic bronchitis pulmonary emphysema with infection
33. Capillary bronchitis
34. Upper respiratory tract infection
35. Community acquired pneumonia
36. Diseases in both lungs for examination
37. Neonatal fever
38. Neonatal pneumonia
39. Neonatal upper respiratory tract infection
40. Inflammatory damage to the lungs
41. Asthmatic bronchial pneumonia in infants
42. Bronchopneumonia in infants
43. Bronchitis in infants
44. Right lung vesicles with infection
45. Diseases in right upper lung for examination
46. Bronchopneumonia
47. Lobular pneumonia
48. Bronchiectasis with infection
49. Bronchitis
50. Severe pneumonia
51. Obstructive pneumonia
52. Pneumonia in left/right lung
53. Left opaque lung for examination
54. Left lung cancer with obstructive pneumonia
55. Left lung shadow for examination
56. Left pneumonia with hemoptysis
